# Analysis of Occupational Stress and Its Relationship with Secretory Immunoglobulin A in the Xinjiang Plateau Young Military Recruits

**DOI:** 10.1155/2020/8695783

**Published:** 2020-04-02

**Authors:** Ning Tao, Hengqing An, Jianjiang Zhang, Yuanyue Zhang, Lu Jin, Lei Xu, Jiwen Liu, Xinjuan Xu

**Affiliations:** ^1^College of Public Health, Xinjiang Medical University, Urumqi 830011, China; ^2^Clinical Post-Doctoral Mobile Stations, Xinjiang Medical University, Urumqi 830011, China; ^3^The First Affiliated Hospital, Xinjiang Medical University, Urumqi 830011, China; ^4^Public Health and Preventive Medicine Post-Doctoral Mobile Station, Xinjiang Medical University, Urumqi 830011, China; ^5^Psychological Medicine Center, The First Affiliated Hospital of Xinjiang Medical University, Xinjiang Medical University, Urumqi, China

## Abstract

**Background:**

With the continuous improvement of the modernization of the Chinese military and the major adjustments made by the state to the recruitment policy, the newly recruited military undergone multiple pressures such as targeted high-intensity military training and environmental changes. The mental health of military has become a crucial factor of improving the fighting capacity effectiveness of the troops.

**Objectives:**

To explore occupational stress of young recruits in the Xinjiang plateau environment during their basic military training period and analyze the relationship between occupational stress and secretory immunoglobulin A (sIgA) levels.

**Methods:**

Using multistage stratified cluster random sampling, 625 recruits stationed at Xinjiang plateau command in 2014 were enrolled as subjects. Occupational stress was assessed by the Occupational Stress Inventory Revised Edition (OSI-R). sIgA in saliva was quantified by enzyme-linked immunosorbent assay. The resulting data were analyzed using descriptive statistics, nonparametric tests, and correlation analysis.

**Results:**

Based on demographic characteristics, occupational stress was higher in the urban group than the rural group, coping ability for stress was greater in individuals who were students before joining the army than nonstudents, occupational stress was higher in smokers than nonsmokers, and coping ability for stress was higher in nonsmokers than in smokers (all *P* < 0.05). Being an only child, educational level and age were not significantly related to occupational stress scores (*P* > 0.05). Salivary sIgA level was higher in the high occupational stress group than in the low stress group (*P* < 0.01). Salivary sIgA was positively correlated with scores on the occupational role and personal strain questionnaires (*r*_*s*_ = 0.229, *r*_*s*_ = 0.268, *P* < 0.01).

**Conclusion:**

Demographic characteristics influenced occupational stress among young recruits in cold and high-altitude area. Further, there were some relationships between occupational stress and salivary sIgA in young military recruits.

## 1. Introduction

The Xinjiang plateau military region, the frontier of China's western strategic direction, is the entire territory of the Xinjiang Uygur Autonomous Region and the Ali area of the Tibet Autonomous Region. The garrison area bordering nine countries is >1.9 million km^2^, and its border line is >6,000 km long. The garrison is widely located in the plateau, desert, and snow mountains. It has the highest altitude of 5,300 m above sea level. In addition, it has the “Shenxian Bay” post, the lowest grassroots company in the Turpan Basin, which is the lowest in the winter, the “Hongshanzui” frontier guard company with extremely cold winter, and the “HuoZhou” combat troops with hot summer temperatures reaching 47°C in summer. The main characteristics of the environment of the Xinjiang plateau are cold climate, gradual temperature decrease with increase in altitude, low oxygen content, and strong ultraviolet radiation. This harsh natural environment along with arduous combat tasks has a potential impact on the physical and mental health of soldiers.

Since the financial crisis in 2008, many countries have suffered from severe economic recession and there has been an employment problem. Under the special background of the lingering after effects of the economic crisis, more and more young people are under pressure from various factors such as family economy, employment, and higher education [1]. In 2009, China made a major adjustment to the conscription policy and recruited young people with higher education enrollment, which provided a new direction to solve the problems of employment [2]. Different from other countries (such as South Korea), China adopted the conscription system that combines conscription with volunteer military service [3]. The implementation of the new military service law has included the college students who were originally listed as deferred enlistment in the range of enlistment, which makes more and more college students and graduates to enlist as a new choice for employment problem.

The military is a special occupational setting that often involves dangerous, emergency, and mandatory tasks. Military employees are considered to be a group with great physical and mental health damage [4]. In an army environment that advocates courage, tenacity, and not fearing difficulties, the mental health of soldiers can be easily neglected to some extent. Soldiers need to maintain high combat effectiveness, which is a special professional characteristic along with a strong tension and stimulation. Military training, special military operation, environment, and actual combat experience greatly influence soldiers' mental health.

At present, men and women who have reached the age of 18 up to 23 can be enlisted for active service according to the needs of the armed forces and on a voluntary basis [5]. Most of the Chinese military's enlisted age is around 18 years or so, the rapid development stage of the body, mind, and self-consciousness makes majority of young people choose to go to college, leaving only a few who choose to join the army at the high school or university stage. The training period of the new recruits is the beginning of their military career, the main workload of which is strict training and assessment. With the continuous improvement of the modernization of the Chinese military, the requirements for the military's quality are also constantly increasing. Massive environmental impact, strict discipline constraints, high intensity military training, and remaining competitive within the various military forces, young military forces around the world young military recruits often end up having high levels of stress. High occupational stress has an important impact on their physical and mental health [6].

Psychological scales, a general survey method, are commonly used to evaluate occupational stress at present [7, 8]. In addition to being closely related to psychological indicators, occupational stress is related to changes in physiological indicators [9], in the systematic view of bodily function, human physiology, and psychology interact. As an important barrier against infection in the upper respiratory tract, salivary secretory immunoglobulin A (sIgA) can be used as a marker of the overall state of the immune system [10]. However, the relationship between psychological stress and level of salivary sIgA at home and abroad are inconsistent [11, 12].

However, the relationship between recruits' occupational stress and saliva sIgA has not previously been reported in the Xinjiang plateau environment. Therefore, by investigating newly enlisted soldiers in the Xinjiang army in 2014, the present study aimed to combine noninvasive physiological indicators and the occupational stress scale to better understand occupational stress during the training period.

## 2. Object and Method

### 2.1. Objects

Adopting a multistage stratified random sampling method, 625 recruits from a military unit in the Xinjiang plateau military region were selected in October 2014. The inclusion criteria were (1) new recruits enrolled in September 2014 and (2) completion of medical examinations before and after enlistment conducted according to the Chinese people's liberation army physical examination standards to exclude any psychological or physiological diseases affecting service. All subjects were aware of the purpose and possible consequences of the study. The participants received no financial reward. This study was approved by the Medical Ethics Committee of Xinjiang Medical University and all participants provided written informed consent.

### 2.2. Method

#### 2.2.1. Demographic Characteristics Survey

An ad hoc questionnaire was used to collect personal information including date of birth, educational level, current smoking status, residence, and job before enlistment.

#### 2.2.2. Occupational Stress Survey

Occupational stress was assessed using the Occupational Stress Inventory Revised Edition (OSI-R) [13]. This instrument comprises three subscales: Occupational Role Questionnaire (ORQ), Personal Strain Questionnaire (PSQ), and Personal Resources Questionnaire (PRQ). ORO assesses role overload, role insufficiency, role ambiguity, role boundary, responsibility (R), and physical environment. PSQ assesses vocational strain, psychological strain (PSY), interpersonal strain, and physical strain (PHS). PRQ assesses recreation (RE), self-care, social support (SS), and rational/cognitive ability. Each subitem contains 10 items, resulting in a total of 140 items. Responses were recorded using a 5-point Likert scale. The higher the scores on the occupational task and stress response questionnaires, the higher was the degree of stress; the higher the scores on the coping resources questionnaires, the stronger was the ability to cope with stress [14]. As recruit training is essentially a form of “work,” it is reasonable to use OSI-R to measure stress during this time. The reliability (Cronbach's coefficient) of the ORQ, PSQ, and PRQ subscales of OSI-R were 0.722, 0.857, and 0.816, respectively, indicating good reliability.

#### 2.2.3. Saliva Samples

Subjects were instructed to not consume any food or drink before sample collection. The subjects sat quietly, tilted their heads forward, and opened their mouths slightly. Saliva was secreted naturally and collected in a sterile sampling cup. Sample volumes ranged from 2 to 5 ml [15, 16]. After collection, saliva samples were centrifuged (3500 RPM × 5 min) and frozen at ﹣86°C until analysis.

#### 2.2.4. Quantification of Salivary sIgA

Salivary sIgA was quantified using the human sIgA enzyme-linked immunosorbent assay (ELISA) kit following the manufacturer's instructions (Shanghai Jianglai Biological Co., Ltd., batch number 201412). Other instruments used include a Bio-Rad microplate reader, pipette (Dalong Medical Equipment Co., Ltd.), electrothermal thermostatic incubator (Shanghai Jinghong Experimental Equipment Co., Ltd.), and microoscillator (Jintan Medical Instrument Factory). The absorbance of the control, samples, and blank were measured at 450 nm wavelength using a microplate reader. The detection limit of sIgA was 1.5 *μ*g/ml.

### 2.3. Quality Control

The experimenters fully explained the purpose of the study, answered any questions, emphasized the anonymous nature of the study, obtained subjects' trust, confirmed voluntary participation, provided accurate information, administered the questionnaire on the spot, and then collected the completed questionnaire.

Saliva was collected after the questionnaire was completed and the saliva sample number corresponded to the questionnaire number. After the experiment, all data were recorded using the EpiData 3.1 software. Two independent investigators verified the accuracy of data entry.

### 2.4. Statistical Analysis

Statistical analysis was performed using the SPSS 16.0 software. First, a normality test was performed. For a nonnormal distribution, data were described by the median (*M*) as well as the 25th and 75th percentiles (*P*_25_, *P*_75_), and Mann–Whitney *U* test was used to analyze differences in occupational stress between demographic groups. Based on ORQ, PSQ, and PRQ scores, subjects were divided into high and low stress groups: subjects with scores greater than or equal to the mean score were assigned to the high subgroup, while with scores below the mean were assigned to the low subgroup [17]. The relationship between occupational stress and salivary sIgA content was analyzed by the Mann–Whitney *U* test. Spearman's correlation coefficient was used to analyze the relationship between occupational stress and salivary sIgA content. The level of statistical significance was set at *P* = 0.05 for a two-tailed test.

## 3. Results

### 3.1. Participant Characteristics

Of the total 625 questionnaires distributed, 623 were recovered. Some questionnaires were excluded because of incomplete information or logical errors, resulting in 597 (95.5%) questionnaires used for analysis. The subjects were all unmarried males with an average age of 18.75 ± 1.34. Of the participants, 152 (25.46%) were from urban areas, 445 (74.54%) were from rural areas, 121 (20.27%) were an only child, 507 (84.92%) had an education level below senior high school, 90 (15.08%) had an education level above junior college, 346 (57.96%) were students before enlistment, 251 (42.73%) were not students before enlisting, 189 (31.66%) were current smokers, and 461 (77.22%) were younger than 20 (Table 1).

### 3.2. Relationship between Demographic Characteristics and Occupational Stress

The median (*P*_25_, *P*_75_) ORQ score was 115.00 (99.00, 130.50) with a range of 67.00–188.0. The median (*P*_25_, *P*_75_) PSQ score was 71.00 (59.00, 87.00) with a range of 41.00–151.00. The median (*P*_25_, *P*_75_) was 140.00 (125.00, 155.00) with a range of 71.00–196.00. The ORQ and PSQ scores of the urban group were higher than those of the rural group; the PRQ scores of individuals who were students before enlistment were higher than those of nonstudents; and the ORQ and PSQ scores smokers were higher than those of nonsmokers, while PRQ scores of nonsmokers were lower than those of nonsmokers (all *P* < 0.05). There was no significant difference in OSI-R subscale scores among new recruits related to being only children, educational level, or age (all *P* > 0.05). These results are summarized in Table 1.

### 3.3. Comparison of Different Occupational Stress Levels and Saliva sIgA Levels

The median (*P*_25_, *P*_75_) salivary sIgA level was 14.11 (10.46–18.29) *μ*g/ml. The comparison of sIgA levels between high and low OSI-R subscale groups showed that the sIgA content in the saliva of high-scoring ORQ and PSQ groups was higher than that of low-scoring groups, and the difference was significant (*P* < 0.01), while the PRQ level and salivary sIgA content changed; however, there was no significant difference (*P* > 0.05), as shown in Table 2.

### 3.4. Correlation between Occupational Stress and Salivary sIgA Content

Spearman's correlation analysis showed that salivary sIgA content was weakly positively correlated with ORQ and PSQ scores (*P* < 0.01), but there was no significant correlation with PRQ scores (*P* > 0.05), as shown in Table 3.

## 4. Discussions

Soldiers stationed in the high and cold area of the Xinjiang plateau have to deal with this special ecological environment all year long, which makes them suffer from higher occupational stress. Under occupational stress for a long time, their physical and mental health status are not optimistic. Enlistment is an important stage of one's career. New recruits must break away from the established lifestyle and social relations, enter a new professional environment, and face a new job. The relatively closed, arduous, and tense training life in the army is stressful for many young soldiers who have just stepped into the barracks [18].

Our survey results showed that in the Xinjiang plateau, occupational stress was higher in the new recruits in the urban group than in the rural group; the coping ability of individuals who were students before enlistment was higher than that of nonstudents; the occupational stress level of smokers was higher than that of nonsmokers; and the coping ability of smokers was lower than that of nonsmokers. Thus, demographic characteristics have different effects on occupational stress among new recruits. There are several possible reasons for this: (1) residence: people from an urban environment feel more pressure because of the faster pace of urban life compared to life in rural China. In addition, because of the greater development of urban infrastructure, recruits from the city who enter the military camp might feel more discomfort, leading to a higher level of occupational stress. (2) Identity before enlisting: due to formal education in schools, interpersonal relationships, and motivation, students may have a better sense of obedience after enlistment and adapt to the tense and regimented life of the army quickly, and thus cope better with occupational stress. By contrast, working or unemployed groups have relatively complex contacts, different experiences, and diverse motivations for enlistment. Their perspectives and positions may affect their ability to cope with occupational stress. (3) Smoking: an unhealthy lifestyle, such as smoking, in the army is a noteworthy phenomenon. Foreign military surveys show that the proportion of mental health disorders among tobacco-dependent populations is higher [19]. This study also suggests that smoking may be associated with occupational stress.

At present, occupational stress scales and related questionnaires are typically used to evaluate occupational stress [20, 21]. There are few studies on occupational stress that include physiological indicators. Although psychological scales are commonly used to evaluate occupational stress, it is undeniable that the results are greatly influenced by subjective factors. Occupational stress, besides being closely associated with the human psychology indicator, is related to human physiological indicators involving the hypothalamus-pituitary-adrenal axis [22], immune system [23], etc. Therefore, it is appropriate to combine physiological indicators with questionnaires to study occupational stress. Various objective physiological indicators have been used to assess occupational stress by scholars in China and abroad, typically using blood samples. However, collecting blood may be an additional stressor; therefore, it may be a confounding factor that could influence the outcome of the experiment. Further, the amount of blood collected is limited, and it is not possible to collect blood for a long time. By contrast, saliva is easy to collect, noninvasive, and has high cost-effectiveness, which has aroused great research interest.

Saliva sIgA plays an important role in local anti-infection, which is closely related to the immune function of the body. According to the results of the present study, in the Xinjiang plateau military region, the high degree of occupational stress of the recruits is associated with an increase in saliva sIgA levels. This shows that the body's immune function is associated with occupational stress. This is likely to be the case of the young soldiers in boot training who start a new career. Because of the tremendous changes in the environment, the body's immune function is affected. This can easily cause bacterial and viral infections. This leads to an increase in sIgA synthesis and secretion in the oral mucosa. In addition, the results suggest that the short-term increase in sIgA synthesis may be an “alert” signal for the body to adapt to occupational stress. Several studies have suggested different changing trends in psychological stress and human sIgA content. Long-term chronic psychological stress can lead to decreased salivary sIgA content [12], whereas acute stress leads to increased salivary sIgA content [24], consistent with the findings of the present study.

This study preliminarily analyzed the relationship between the Xinjiang plateau military region recruits' occupational stress and sIgA content in saliva, which has some limitations. Firstly, cross-sectional epidemiological research methods cannot determine an exact causal relationship or an exact correlation mechanism; rather, it can only provide clues for further cohort studies. Secondly, all respondents were unmarried males aged 20 or so in Xinjiang, China, and no females were included. These demographic characteristics are quite different from those of the general population. Thirdly, occupational stress was assessed by the OSI-R and saliva sIgA was quantified by ELISA, which had no clinical diagnostic value. Lastly, it was impossible to analyze dynamic changes in occupational stress and sIgA content because saliva was collected at a single time point.

Despite these shortcomings, this study helps deepen the understanding of the relationship between the Xinjiang plateau military region recruits' occupational stress and physiological indicators. Due to its reliability and the simplicity of sample collection, storage, and analysis, further study of the relationship between salivary sIgA and occupational stress in soldiers is warranted.

## 5. Conclusion

The cold climate, gradual temperature decrease with increase in altitude, low oxygen content, strong ultraviolet radiation of the Xinjiang plateau, and arduous combat tasks have a potential impact on the physical and mental health of soldiers. Environmental changes along with discipline and restrictions have an impact on occupational stress level after enlistment of the soldiers. Salivary sIgA is positively correlated with occupational role and personal strain. Thus, salivary sIgA can be used to explore occupational stress.

## Figures and Tables

**Table 1:** Influence of demographic characteristics on occupational stress (*n* = 597).

Group	Number	Proportion	ORQ	PSQ	PRQ
Registered residence					
Urban	152	25.46	117.00 (100.00, 137.75)	75.50 (60.00, 95.00)	137.00 (121.25, 154.00)
Rural	445	74.54	114.00 (98.00, 129.00)	69.00 (58.00, 84.00)	140.00 (125.50, 156.00)
*Z*_1_			-1.998	-2.133	-1.358
*P*_1_			0.046	0.033	0.175
Singe child					
Yes	121	20.27	116.00 (98.25, 133.00)	73.00 (60.00, 87.75)	140.50 (125.25, 154.00)
No	476	79.73	114.00 (99.00, 130.00)	70.00 (58.00, 87.00)	139.50 (125.00, 156.00)
*Z*_2_			-0.331	-1.104	-0.236
*P*_2_			0.741	0.269	0.814
Education level					
High school or above	507	84.92	115.00 (99.00, 131.00)	71.00 (59.00, 88.00)	139.00 (124.00, 155.00)
College or above	90	15.08	116.00 (97.75, 129.25)	69.50 (57.00, 83.25)	141.00 (130.00, 157.00)
*Z*_3_			-0.44	-0.43	-1.115
*P*_3_			0.66	0.667	0.265
Social status before enlisting					
Student	346	57.96	114.00 (99.00, 130.25)	69.00 (57.00, 86.00)	142.00 (126.75, 157.00)
Not student	251	42.04	116.00 (98.00, 131.00)	73.00 (60.00, 89.00)	137.00 (123.00, 151.00)
*Z*_4_			-0.558	-1.705	-2.323
*P*_4_			0.577	0.088	0.02
Smoker					
Yes	189	31.66	119.00 (100.00, 134.00)	73.00 (61.00, 94.50)	137.00 (120.00, 152.00)
No	408	68.34	113.00 (98.00, 129.00)	69.00 (58.00, 84.00)	141.00 (126.00, 157.00)
*Z*_5_			-2.096	-2.782	-2.379
*P*_5_			0.036	0.005	0.017
Age					
≥20	136	22.78	114.00 (99.00, 129.00)	71.00 (58.50, 86.50)	140.00 (125.00, 156.00)
<20	461	77.22	120.00 (98.00, 136.75)	69.00 (59.00, 90.50)	138.00 (124.00, 154.00)
*Z*_6_			-1.445	-0.147	-0.791
*P*_6_			0.148	0.883	0.429

The values of *Z*_1_ and *P*_1_ were the results of comparing occupational tasks, stress response, and coping resources scores among groups with different family locations; the values of *Z*_2_ and *P*_2_ were the results of comparing occupational tasks, stress response, and coping resources scores among groups with different family sizes; the values of *Z*_3_ and *P*_3_ were the results of comparing occupational tasks, stress response, and coping resources scores among groups with different educational levels; the values of *Z*_4_ and *P*_4_ were the results of comparing occupational task, stress response, and coping resource scores among different preenlistment status groups; the values *Z*_5_ and *P*_5_ were the results of comparing occupational task, stress response, and coping resource scores between smoking and nonsmoking groups; and the values *Z*_6_ and *P*_6_ were the results of comparing occupational task, stress response, and coping resource scores among different age groups.

**Table 2:** Comparison of salivary sIgA and occupational stress.

Group	Number	Proportion (%)	Salivary sIgA	*Z*	*P*
			(*M* (*P*_25_, *P*_75_) (*μ*g/ml))		
Occupational role				-4.027	0.001
Low	314	52.6	12.40 (11.38-18.29)		
High	283	47.4	15.32 (11.67-19.55)		
Personal strain				-3.263	0.001
Low	353	59.13	12.28 (8.69-15.04)		
High	244	40.87	14.65 (10.94-18.99)		
Personal resources				-1.879	0.060
	296	49.58	14.75 (11.38-18.29)		
	301	50.42	13.05 (9.96-18.14)		

**Table 3:** Correlation of occupational stress and salivary sIgA (Spearman's correlation coefficient).

Occupational stress subscale	Salivary sIgA level (*μ*g/ml)	
	*r*_*s*_	*P*
ORQ	0.229	0.001
PSQ	0.268	0.001
PRQ	-0.124	0.054

## Data Availability

The (SAV) data used to support the funding of this study are restricted in order to protect the participant privacy. Data are available from Jiwen Liu (liujiwen@xjmu.edu.cn) for researchers who meet the criteria for access to confidential data.
